# Feasibility of novel four degrees of freedom capacitive force sensor for skin interface force

**DOI:** 10.1186/1475-925X-11-90

**Published:** 2012-11-27

**Authors:** Chisato Murakami, Yusuke Ishikuro, Makoto Takahashi

**Affiliations:** 1 Division of Biomedical Systems Engineering, Graduate School of Information Science and Technology, Hokkaido University, Hokkaido, Japan; 2 Hokkaido Electric Power Co., Inc., Hokkaido, Japan

**Keywords:** Capacitive sensor, Force sensor, Four DOF, Skin surface, Capacitance

## Abstract

**Background:**

The objective of our study was to develop a novel capacitive force sensor that enables simultaneous measurements of yaw torque around the pressure axis and normal force and shear forces at a single point for the purpose of elucidating pressure ulcer pathogenesis and establishing criteria for selection of cushions and mattresses.

**Methods:**

Two newly developed sensors (approximately 10 mm×10 mm×5 mm (10) and 20 mm×20 mm×5 mm (20)) were constructed from silicone gel and four upper and lower electrodes. The upper and lower electrodes had sixteen combinations that had the function as capacitors of parallel plate type. The full scale (FS) ranges of force/torque were defined as 0–1.5 N, –0.5-0.5 N and −1.5-1.5 N mm (10) and 0–8.7 N, –2.9-2.9 N and −16.8-16.8 N mm (20) in normal force, shear forces and yaw torque, respectively. The capacitances of sixteen capacitors were measured by an LCR meter (AC1V, 100 kHz) when displacements corresponding to four degrees of freedom (DOF) forces within FS ranges were applied to the sensor. The measurement was repeated three times in each displacement condition (10 only). Force/torque were calculated by corrected capacitance and were evaluated by comparison to theoretical values and standard normal force measured by an universal tester.

**Results:**

In measurements of capacitance, the coefficient of variation was 3.23% (10). The Maximum FS errors of estimated force/torque were less than or equal to 10.1 (10) and 16.4% (20), respectively. The standard normal forces were approximately 1.5 (10) and 9.4 N (20) when pressure displacements were 3 (10) and 2 mm (20), respectively. The estimated normal forces were approximately 1.5 (10) and 8.6 N (10) in the same condition.

**Conclusions:**

In this study, we developed a new four DOF force sensor for measurement of force/torque that occur between the skin and a mattress. In measurement of capacitance, the repeatability was good and it was confirmed that the sensor had characteristics that enabled the correction by linear approximation for adjustment of gain and offset. In estimation of forces/torque, we considered accuracy to be within an acceptable range.

## Background

For prevention of pressure ulcers, information on the amount of forces and the directions of forces is important for the setup of depressurization from the skin surface and for elucidation of the mechanism in pressure ulcer pathogenesis. Pressure ulcer is ischemic tissue necrosis. The main cause of pressure ulcers is locally sustained forces applied to the skin surface. The stress generated by the forces is intensely focused on bony prominences (e.g., sacrum and ischium) and promotes the development of pressure ulcers. Japan’s population is now rapidly aging. In 2010, the population of Japan was approximately 128,057,000, with 29,484,000 people over the age of 65 years. The proportion of people over 65 years of age accounted for 23% of the total population [1], and it is predicted that the proportion of elderly people will continue to increase. Since the proportion of people confined to bed will also increase, particular disorders (such as pressure ulcers) that develop in such a condition will also increase and will become an important social issue in the aged society.

Studies on pressure ulcers have been multidisciplinary studies including fields such as medical science, nursing [2] and engineering. Studies of relation between pressure ulcers and forces of pressure and shear directions has been examined for clinical applications. Salcido et al. reported skin responses of various animal models to pressure and friction [3]. Animal models are used for experimental observation of the skin directly above a bony prominence because such observation is difficult in human subjects. Animal models have been used not only in histological studies but also for assessment of wound care products. Ohura et al. validated the mechanical property of a commercially available wound dressing using a skin model with a bony prominence consisted of porcine skin and force sensors [4]. One of the sensors is a pneumatic detection apparatus (Predia, Molten) that is flexible and thin and has the ability to measure pressure and shear forces simultaneously. Measurements of forces on the skin model indicated that dressing materials can reduce shear force. The amount of force applied to the skin and the amount of stress produced by the force have been shown to be associated with cushion and mattress materials and body position [5]. The sensor was also used for quantification of the effectiveness of a cushion and a mattress. Akins et al. measured interface shear stress, interface pressure, and horizontal stiffness of commercially available wheelchair seat cushions [6]. Information on a cushion’s effectiveness is necessary for selection of appropriate patient-specific cushion. Pressure and shear characteristics of various cushion materials were determined by a loading apparatus using a rigid cushion loading indenter of buttocks configuration. As stated above, recent studies related to pressure ulcers have focused on shear force in addition to pressure for reduction of these forces.

**Figure 1:**
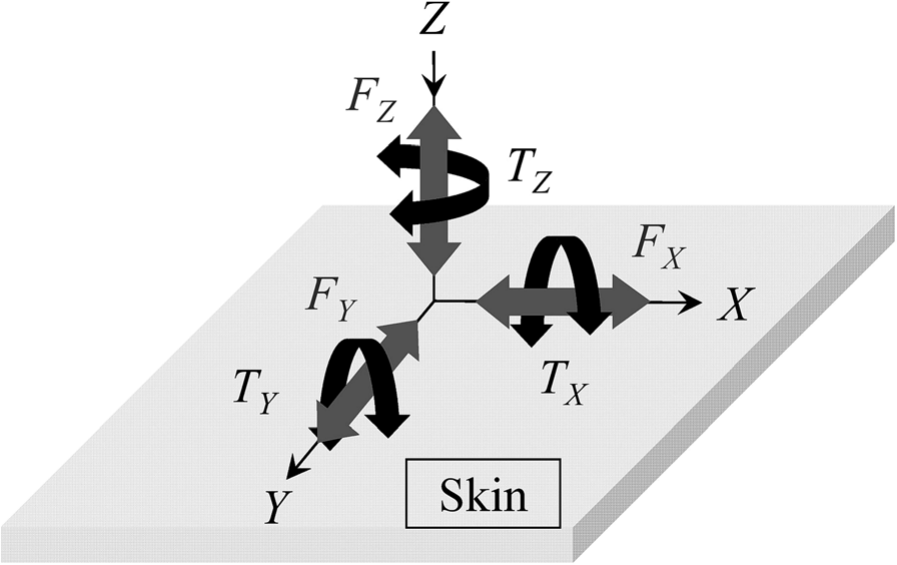
**Force and torque components of six DOF on the skin surface.** In the *XY* plane as the skin surface, the force component of the vertical axis is normal force *F*_Z_ and the force components of horizontal axes are shear forces *F*_X_ and *F*_Y_. The torque components around the axes are roll, pitch and yaw (*T*_X_, *T*_Y_ and *T*_Z_).

Components of force applied to the skin surface consist of six degrees of freedom (DOF). When the skin surface is defined as the *XY* plane in a Cartesian coordinate system, force components in *X*, *Y* and *Z* axes directions are denoted as shear forces *F*_X_ and *F*_Y_ and normal force *F*_Z_, respectively (Figure 1). Roll, pitch, and yaw torques (*T*_X_, *T*_Y_ and *T*_Z_) are represented in rotation directions around *X*, *Y*, and *Z* axes. Recent studies on pressure ulcers have also focused on yaw torque [7], which can occur with postural change and weight shift, in addition to normal force and shear forces. However, quantitative evaluation of yaw torque has not been performed because there is no sensor device for application to the skin surface.

Various six DOF force sensors have been developed for industrial applications. In the field of robotics, results of studies on the development of a multi DOF sensor have been reported. Chao et al. reported a strain gauge-based six DOF force transducer for the detection of wrist force [8]. These robotic force sensors require heavy duty construction. However, a flexible and thin structure is desirable for a sensor for measuring force applied to the skin surface [9]. In research related to a tactile sensor, a flexible, thin, and small sensor for detecting three DOF (normal force and shear forces) has been developed by micromachining technology. Wang et al. developed a silicon sensor using four piezoresistors [10]. The forces were calculated by the combination of several piezoresistors. The voltage of each resistor was measured in the calibration ranges of compressive force (0–30 N) and shear force (5–40 N). Hwang et al. developed a device for application not only as a tactile sensor but also as a ground reaction force sensor for balance control in humanoid robots [11]. Each element of the sensor was constructed from polyimide and polydimethylsiloxane substrate and there were four thin-film metal strain gauges for flexibility. The operation of one element was confirmed by the ranges of normal load (0–4 N) and shear force (0–1.5 N). The operation was also confirmed in 8×8 sensors and a ground reaction force sensor. Many of these sensors have the ability to detect three DOF forces. In the forementioned studies related to force sensors, although the sensors were developed by using a strain gauge, a capacitive tactile sensor has also been reported. da Rocha et al. developed a capacitive tactile sensor by using one upper electrode, four lower electrodes (aluminum) and flexible dielectric material (rubber) [12]. The forces were calculated by four capacitors constructed from four combinations of upper and lower electrodes. The operation was confirmed by the force as displacement of pressure direction (0–200 μm) and the displacement of shear direction (0–250 μm). Cheng et al. presented a polymer-based capacitive sensing array by polydimethylsiloxane and flexible printed circuit board (FPCB) [13]. In order to solve the problem of vulnerability of the interconnect line between sensing areas, the interconnect line was arranged in the lower substrate side only. One floating electrode and two lower electrodes had the function of a pseudo-parallel-plate capacitive mechanism. The operation as a 4×4 shear sensing array was confirmed by a loaded condition with normal force of 5.5 N and shear force of 3.1 N. One of the problems in the capacitive sensor is small capacitance due to miniaturization of the structure and there is an increase in the rate of stray capacitance. Our desired sensors of two types had different sensing areas of 10 mm×10 mm and 20 mm×20 mm. The 10 mm square sensor can be used for application to the calcaneus, which is the minimum bony prominence in the human body, while the 20 mm square sensor can be used for application to bony prominences that are larger than the calcaneus. Our sensor had four upper electrodes and four lower electrodes as sixteen capacitors. Forces and torque were estimated by the capacitance values in the capacitors. We considered that likely forces and torque are ensured by estimation using several combinations of upper and lower electrodes even if stray capacitance exists in the measurement environment. The capacitive sensor has the advantage of sensitivity being easily changed by selection of the dielectric. An appropriate dielectric was selected for this sensor.

The goal of our study was to develop a novel capacitive force sensor that enables simultaneous measurements of yaw torque around the pressure axis as well as normal force and two orthogonal shear forces at a single point. We describe the fabrication of a prototype sensor, results of measurement of capacitance characteristics and results showing the validity of estimated forces and torque. For the purpose of elucidation of pressure ulcer pathogenesis and establishment of criteria for selection of cushions and mattresses, it is important to determine the direction and amount of forces applied to the affected area of the skin surface. The newly developed sensor has a structure that also enables detection of torque components *T*_X_ and *T*_Y_.

## Sensor theory

### Sensor structure

**Figure 2:**
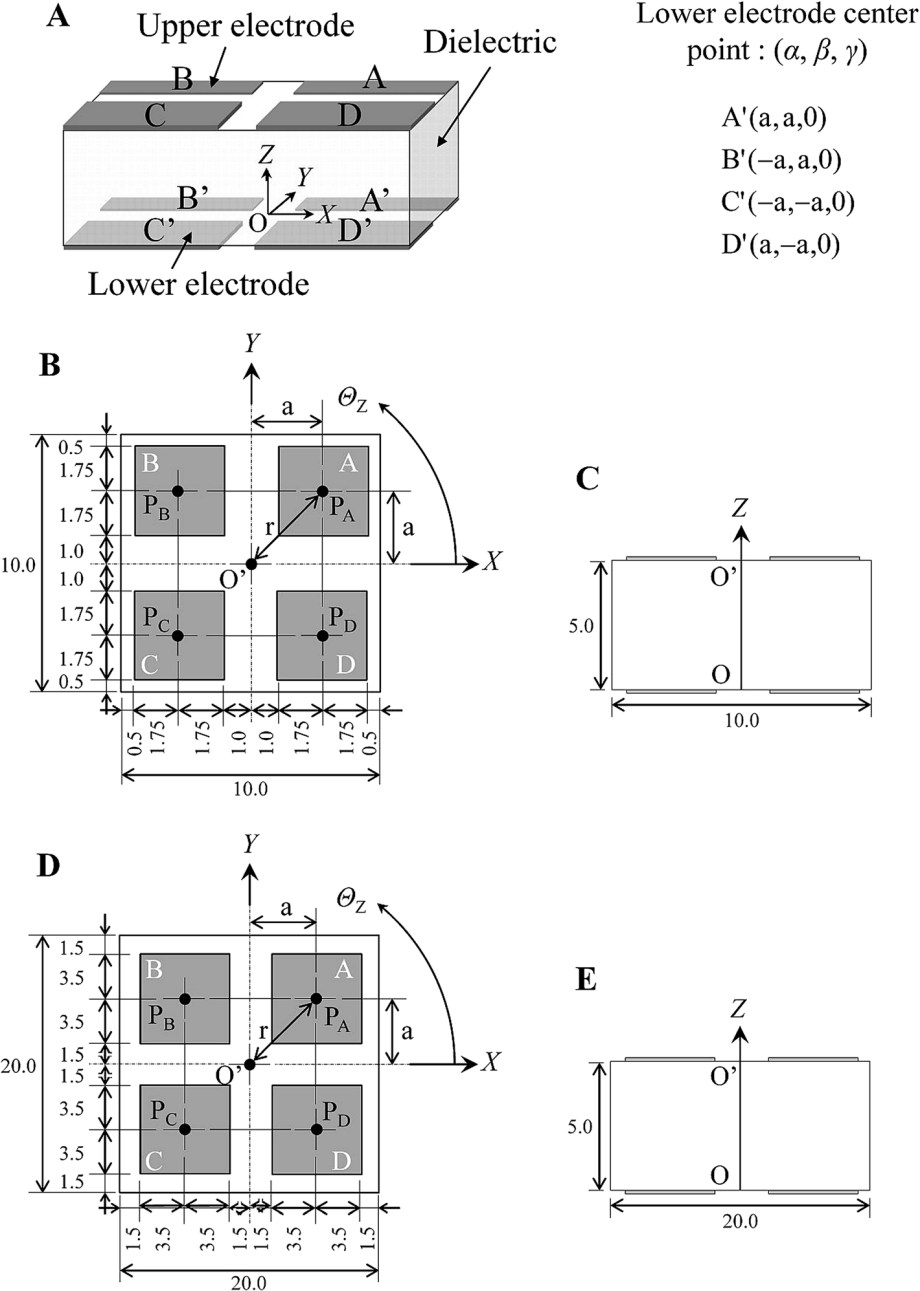
**Structure and coordinate system of the newly developed sensor. A.** Overall view. **B and D.** Top view. **C and E.** Side view. **B and C.** Images of the 10 mm square sensor (a = 2.75 mm). **D and E.** Images of the 20 mm square sensor (a = 5 mm).

The newly developed sensor was constructed from a cubic dielectric and four upper and lower electrodes (Figure 2). The upper electrodes are denoted by A, B, C, and D and the lower electrodes are denoted by A’, B’, C’, and D’ (Figure 2A). There are sixteen combinations of upper and lower electrodes: AA’, AB’, …, and DD’. Each paired electrode has the function of a capacitive parallel plate type. Components of the four DOF were defined as *X* and *Y* in the shear directions, *Z* in the pressure direction and yaw angle *Θ*_Z_ in the rotation direction around the *Z* axis. The origin of the coordinate system O was established as the center point of the lower substrate. The center point of the upper substrate was denoted as O’. (*α*, *β*, *γ*) is the initial position of the lower electrode center point in an unloaded condition. The initial angles of upper and lower electrodes in an unloaded condition are *φ*_A_ = *φ*_A’_ = 45 degrees, *φ*_B_ = *φ*_B’_ = 135 degrees, *φ*_C_ = *φ*_C’_ = 225 degrees, and *φ*_D_ = *φ*_D’_ = 315 degrees. Center points of the electrodes were defined as P_A_, P_B_, …, and P_D’_. Tables 1 and 2 show the desired displacements and force/torque ranges for the 10 mm square sensor and 20 mm square sensor. 

**Table 1:** Displacement and force/torque ranges of the 10 mm square sensor in each direction

**Direction**	**Displacement range**			**Force/torque detection range**		
	**Min**	**Max**	**Unit**	**Min**	**Max**	**Unit**
***X***	−3	3	mm	−0.5	0.5	N
***Y***	−3	3	mm	−0.5	0.5	N
***Z***	0	3	mm	0	1.5	N
***Θ***_**Z**_	−30	30	degree	−1.5	1.5	N·mm

**Table 2:** Displacement and force/torque ranges of the 20 mm square sensor in each direction

**Direction**	**Displacement range**			**Force/torque detection range**		
	**Min**	**Max**	**Unit**	**Min**	**Max**	**Unit**
***X***	−2	2	mm	−2.9	2.9	N
***Y***	−2	2	mm	−2.9	2.9	N
***Z***	0	2	mm	0	8.7	N
***Θ***_**Z**_	−10	10	degree	−16.8	16.8	N·mm

### Calculation of displacement components

When a force is applied to the sensor and the dielectric is deformed, the capacitance changes due to change of the distance between upper and lower electrodes. The amount of capacitance change is different for each capacitor under a loaded condition applied to the sensor. The capacitance value *C* at a capacitor is defined as (1).

(1) $$C=\varepsilon_{0}\varepsilon_{\text{r}}S/d\text{,}$$

where *ε*_0_ is the permittivity of vacuum, *ε*_r_ is the relative permittivity of the dielectric material, *S* is the area of the parallel plate electrode, and *d* is the distance between the upper and lower electrodes and can be calculated by *C*.

The center point of one upper electrode (*x*, *y*, *z*) is determined using three *C* values. When the lower electrode center point (*α*, *β*, *γ*) is defined as the center of a sphere, the equation of a sphere with radius *d* is denoted as (2).

(2) $${\left(x-\alpha \right)}^{2}+{\left(y-\beta \right)}^{2}+{\left(z-\gamma \right)}^{2}=d^{2}\text{.}$$

**Figure 3:**
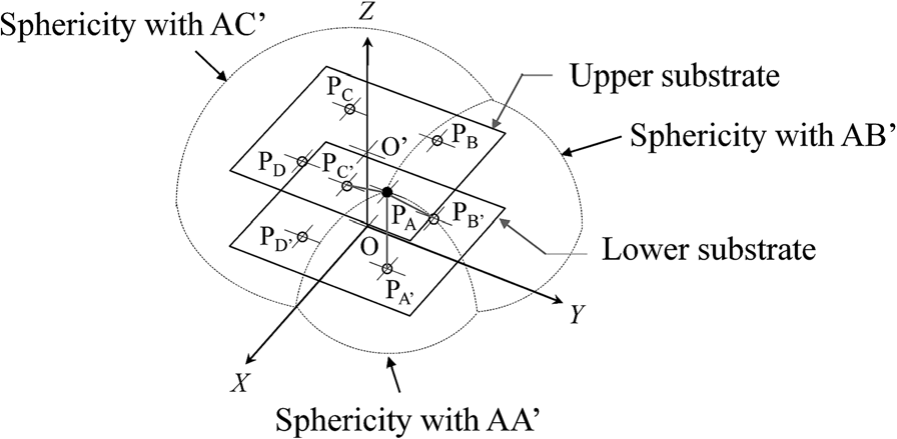
Method for detecting position of the upper electrode A.

The intersection of three spherical surfaces means the position of an upper electrode. For example, the center point P_A_ is calculated from three capacitances in AA’, AB’, and AC’ (Figure 3). P_B_, P_C_, and P_D_ can be calculated by the same method. The averaged positions of *x*, *y*, and *z* in P_A_, P_B_, P_C_, and P_D_ denote the center point of the upper substrate. The displacements *X* and *Y* are the difference in position of the center point of the upper substrate between loaded and unloaded conditions. Displacement *Z* is the difference between initial thickness of the sensor and the averaged position of *z* in P_A_, P_B_, P_C_ and P_D_. If either one substrate has a lean against the other substrate, calculated *z* differs among each upper electrode. Therefore, it was assumed that the upper substrate is parallel to the lower substrate by averaging of *z* of four upper electrodes. Yaw angle *Θ*_Z_ is determined by using a rotation matrix as shown in (3).

(3) $$\left(\begin{array}{c} x_{2}\\ y_{2}\\ z_{2} \end{array}\right)=\left(\begin{array}{ccc} \cos\Theta_{\text{Z}} & -\sin\Theta_{\text{Z}} & 0\\ \sin\Theta_{\text{Z}} & \cos\Theta_{\text{Z}} & 0\\ 0 & 0 & 1 \end{array}\right)\left(\begin{array}{c} x_{1}\\ y_{1}\\ z_{1} \end{array}\right)\text{,}$$

where (*x*_1_, *y*_1_, *z*_1_) is the position of the electrode center point before rotation and (*x*_2_, *y*_2_, *z*_2_) is the position of the electrode center point after rotation. Roll and pitch angles were also detected by the same solution method.

### Calculation of force and torque components

Firstly, the normal and shear strains *ε*, *γ*_*x*_ and *γ*_*y*_ are obtained from the displacements *Z*, *X* and *Y*, respectively. Secondly, the normal and shear stresses *σ*, *τ*_*x*_ and *τ*_*y*_ can be calculated by Hooke’s law using the normal and shear strains and are denoted as *σ* = *Eε*, *τ*_*x*_ = *Gγ*_*x*_, and *τ*_*y*_ = *Gγ*_*y*_. *E* is the elastic modulus and *G* is the modulus of rigidity. The normal and shear forces *F*_Z_, *F*_X_ and *F*_Y_ applied to the sensor were calculated from the stresses *σ*, *τ*_*x*_ and *τ*_*y*_ as (4). The torsion moment as yaw torque *T*_Z_ is defined as (5).

(4) $$F_{\text{Z}}=\sigma A,F_{\text{X}}=\tau_{x}A,F_{\text{Y}}=\tau_{y}A\text{,}$$

(5) $$T_{\text{Z}}=GI_{p}\Theta_{\text{Z}}/l\text{,}$$

where *A* is the cross-sectional area of the pressure direction, *l* is the length of the sensor in the pressure axis under an unloaded condition and *I*_*p*_ is the second moment in a rectangular section. *I*_*p*_ was calculated from WD(W^2^ + D^2^)/12. W is width and D is depth of the rectangular section [14].

## Methods

### Sensor design and fabrication

**Figure 4:**
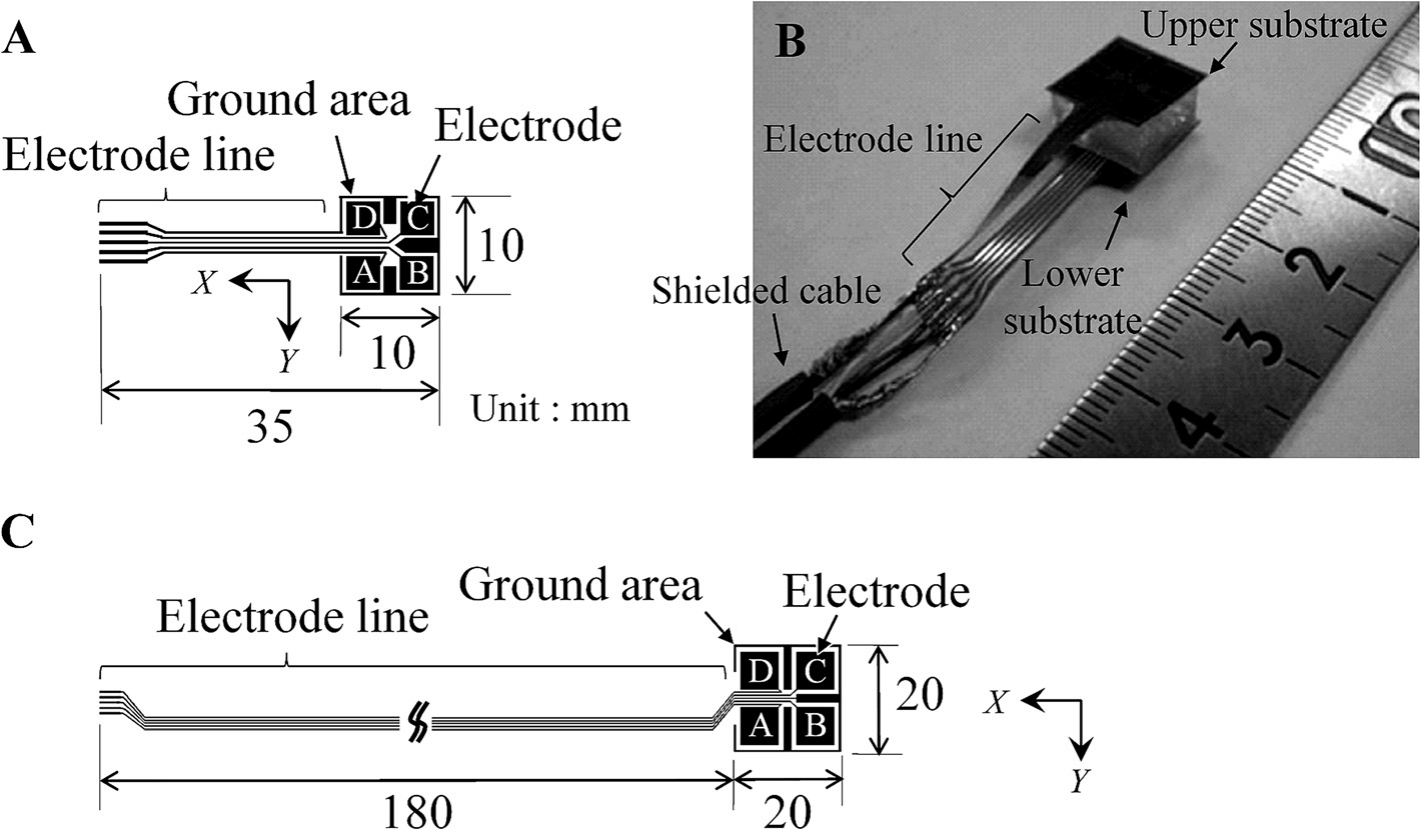
**Electrode pattern of electrodes and electrode lines on FPCB and fabricated sensor. A.** Electrode pattern of the 10 mm square sensor. **B.** Fabricated sensor of the 10 mm square sensor. **C.** Electrode pattern of the 20 mm square sensor.

The sensor was constructed from a cubic dielectric (silicone gel, approximately 10 mm×10 mm×5 mm and 20 mm×20 mm×5 mm) and upper and lower substrates. In an unloaded condition, the initial distance between upper and lower electrodes in AA’, BB’, CC’ and DD’ was *d* = 5 mm. The electrode pattern of the substrate was formulated as shown in Figure 4A (10 mm square sensor) and C (20 mm square sensor). The substrates (about 10 mm×10 mm and 20 mm×20 mm) are each constructed from four electrodes (copper), ground area, and five lines. Electrodes are 3.5 mm×3.5 mm in the 10 mm square sensor and 7 mm×7 mm in the 20 mm square sensor. More detailed dimensions are shown in Figure 2. Four lines were connected to each electrode and one line was connected to the ground area. The pattern was exposed on FPCB (Sunhayato, 1K, Japan). Then the substrate was formed by etching. The lines on the substrates were connected to four core shielded cables for reduction of power noise (approximately 400 mm in the 10 mm square sensor and 300 mm in the 20 mm square sensor). The gel and two substrates were bonded with double-sided tape that had silicone and acrylic pressure-sensitive adhesive. The thickness of the double-sided tape is 0.085 mm. Figure 4B shows the fabricated 10 mm square sensor. Each sensor was positioned between the skin and mattress and was used for the measurement at the heel and elbow by the 10 mm square sensor and at the skin under the sacrum and ischium by the 20 mm square sensor. These regions are common sites of pressure ulcers in dorsal and sitting positions.

Silicone gel as a dielectric material was used in consideration of the utilization purpose, i.e., measurement of force applied to the skin surface. Therefore, we defined the standard of the pressure range to be 200 mmHg (about 26.6 kPa), which is the standard pressure in a seated position. The elastic modulus *E*, creep modulus *RC*, and compression set *CS* of the gel were measured on the basis of JIS K 6254, JIS K 6273, and JIS K 6262. The gel was cut into a 10 mm dice, and ambient temperature of the experimental laboratory was maintained at 23 degrees C. Silicone gel was selected on the basis of two conditions. One is that deformation of gel is strain of 50% in pressure of 200 mmHg. The other is low *RC* that means a small effect in temporal alteration of strain. As a result, the gel was selected for the fabricated sensor (*E* = 25.7 kPa, *RC* = 1.0%, and *CS* = 0.7%). *E* of the 20 mm square sensor was measured using silicone gel of 20 mm×20 mm×5 mm and it was *E* = 54.1 kPa. The rigidity modulus *G* was calculated to be 8.6 kPa (10 mm square sensor) and 18.0 kPa (20 mm square sensor) by *G* = *E* / (2(1 + *ν*)), where *v* is Poisson’s ratio that is defined as 0.5 because silicone gel was considered to be an incompressibility elastic body. The second moment *I*_*p*_ was 1.67×10^–9^ m^4^ (10 mm square sensor) and 26.7×10^–9^ m^4^ (20 mm square sensor). The relative permittivity of the gel was estimated by the characteristics of measured and calculated capacitance in load of the pressure axis direction only. The relative permittivity *ε*_r_ was 4.8.

### Experimental system

**Figure 5:**
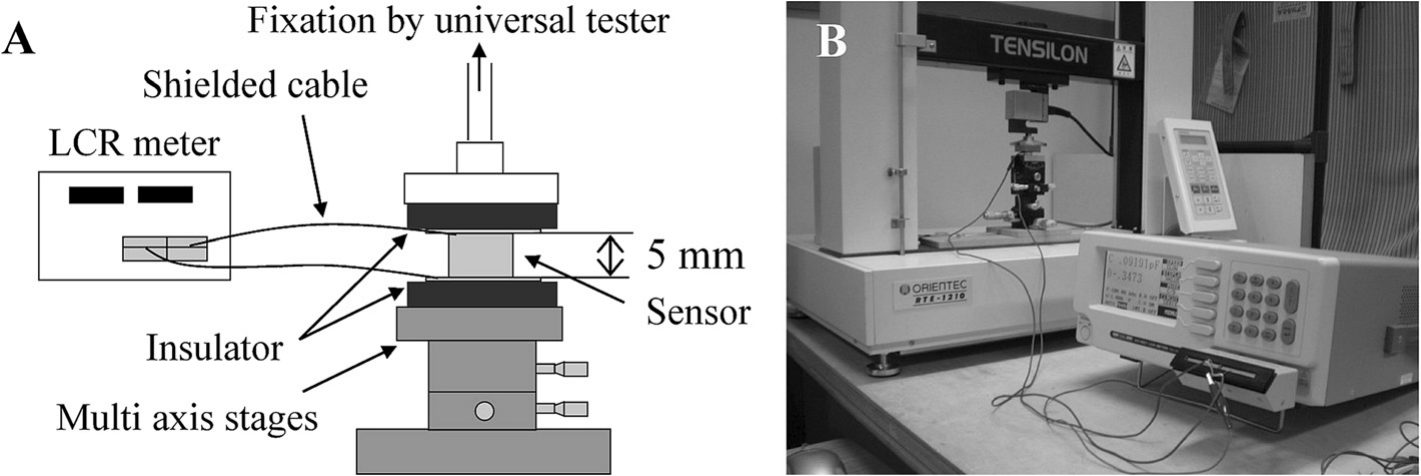
**Measurement system. A.** The measurement system was constructed from multi-axis stages, an LCR meter and a universal tester. **B.** Photograph of capacitance measurement.

The capacitance was measured using a measurement system (Figure 5). The system was constructed from multi-axis stages (XYZθαβ axis stages, SIGMA KOKI), an LCR meter (KC-567, KOKUYO ELECTRIC), and a universal tester (TENSILON, RTE-1210, ORIENTEC). The measurement conditions were voltage source of AC1V, measuring frequency of 100 kHz and measurement time of 896 msec. Displacements as forces of four DOF were applied to the lower surface of the sensor by the multi-axis stages. The capacitance changes of sixteen combinations of upper and lower electrodes were measured by the LCR meter. The standard normal force was measured by the universal tester.

## Results

### Capacitance characteristics

Theoretical capacitance was calculated by (1) when relative permittivity *ε*_r_ is 4.8 and electrode area *S* is 3.5 mm×3.5 mm (10 mm square sensor) and 7 mm×7 mm (20 mm square sensor). Theoretical *d* was defined as (6) from the displacement of four DOF.

(6) $$d=\sqrt{d_{x}^{2}+d_{y}^{2}+d_{z}^{2}}\text{,}$$

where *d*_*x*_ = rcos*φ* – (*X* + rcos*φ* ’), *d*_*y*_ = rsin*φ* – (*Y* + rsin*φ*’), and *d*_*z*_ = 5 – *Z*. r is the distance between the origin O and the center point of the lower electrode and is a constant value (Figure 2B, D). a is 2.75 mm in the 10 mm square sensor and 5 mm in the 20 mm square sensor. *φ’* is the position angle of the electrode in a loaded condition *φ’* = *φ* + *Θ*_Z_. We defined groups of combinations of upper and lower electrodes as FEC (facing electrode combination: AA’, BB’, CC’, and DD’), DEC (diagonal electrode combination: AC’, BD’, CA’, and DB’), and OEC (other electrode combination).

**Figure 6:**
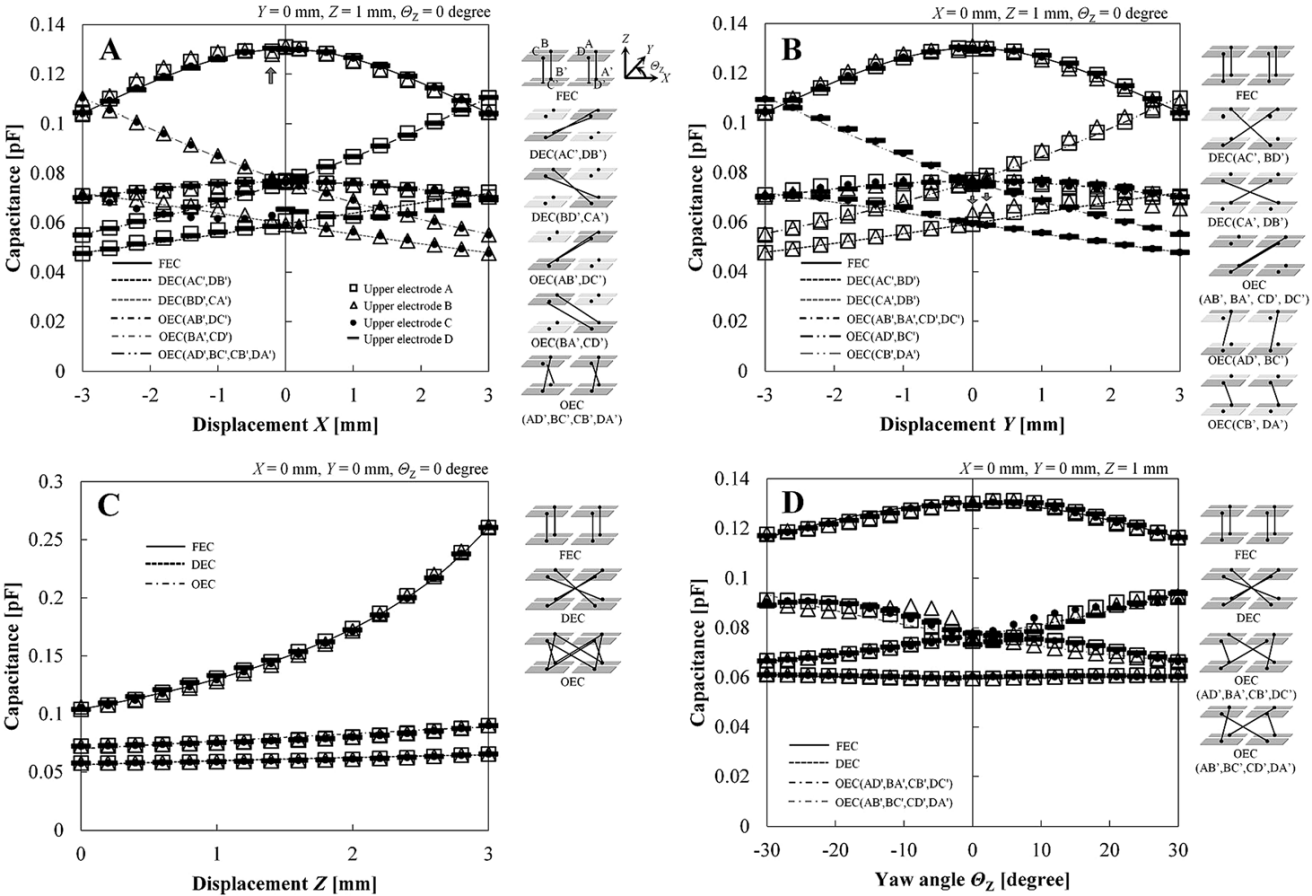
**Characteristics of theoretical and corrected capacitances in each displacement condition (10 mm square sensor). A.** Varied displacement *X*. **B.** Varied displacement *Y*. **C.** Varied displacement *Z*. **D.** Varied angle *Θ*_Z._

In the measurement of capacitance characteristics of the 10 mm square sensor, displacements of the four DOF varied within the ranges shown in Table 1, and the steps were 0.4 mm, 0.4 mm, 0.2 mm and 3 degrees in *X*, *Y*, *Z* and *Θ*_Z_ directions, respectively. Capacitances at displacements *X* and *Y* = 0 mm were also measured along with the above measured points. *Z* was 1 mm and the other two displacements were constant values of zero in the conditions of varied *X*, *Y*, and *Θ*_Z_. The measurement was repeated three times in each displacement condition. The coefficient of variation was calculated by standard deviation in the measurement of three times. The averaged value of the coefficients of variation through all measured points was 3.23%. In the 20 mm square sensor, the measured point was 625 at combinations of four DOF displacements *X* = −2, –1, 0, 1, 2 mm, *Y* = −2, –1, 0, 1, 2 mm, *Z* = 0.5, 0.8, 1.2, 1.6, 2.0 mm, and *Θ*_Z_ = −10, –5, 0, 5, 10, and measurement was carried out only once at each point. The measured capacitance was corrected by linear approximation for adjustment of gain and offset using the theoretical capacitance. The corrected point was three in the condition of varied *Z*. The corrected points in the conditions of varied *X*, *Y* and *Θ*_Z_ directions were three points to positive and negative domains, respectively. In the measurement of capacitances in sixteen capacitors, it was confirmed that the sensor had capacitance characteristics that enabled the correction by linear approximation for adjustment of gain and offset only. However, the method cannot correct displacement within a measurement range without the measured point. Therefore, a different method should be used for the estimation of displacements in arbitrary points without calibration points as shown in the section “Discussion, Capacitance characteristics of the 10 mm square sensor”. Figure 6 shows the characteristics of theoretical and corrected capacitances in the 10 mm square sensor. The horizontal axis is the varied displacement of each direction and the vertical axis is the capacitance. The lines are theoretical values and dots are corrected values. The dots were described by the upper electrode. The figure on the right side of the characteristic graph shows the positional relation of combinations of upper and lower electrodes.

### Calculation of force and torque components

**Figure 7:**
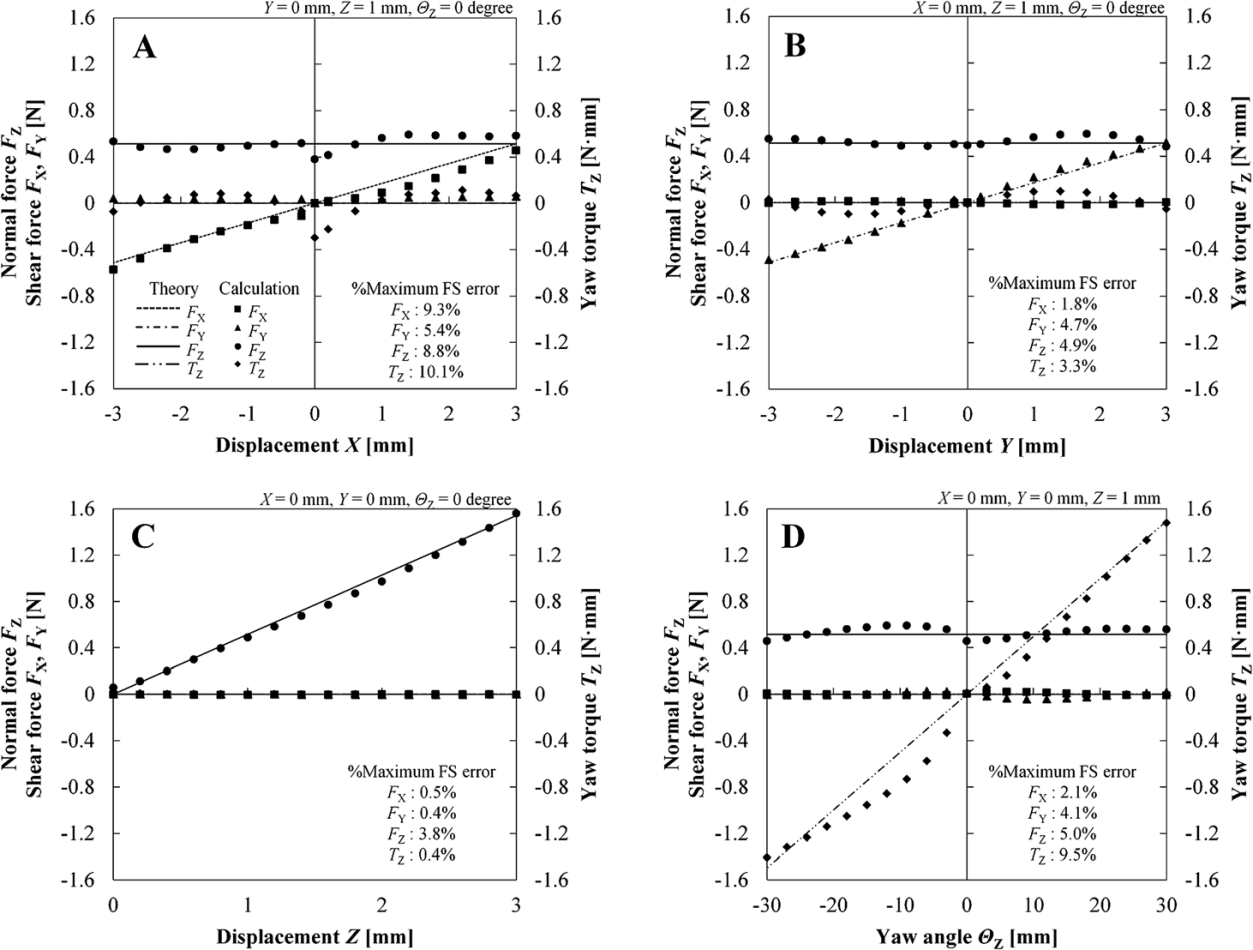
**Characteristics of theoretical and calculated force and torque components in each displacement condition (10 mm square sensor). A.** Varied displacement *X*. **B.** Varied displacement *Y*. **C.** Varied displacement *Z*. **D.** Varied angle *Θ*_Z_.

Displacements in the measured points were estimated by the corrected capacitance, and the force and torque components were calculated by (4) and (5). Figure 7 shows the results of calculated forces and torque corresponding to the measured points in the 10 mm square sensor. The horizontal axis is the varied displacement of each direction and the vertical axis is the forces and torque of four components. Calculated forces and torque were compared with theoretical values calculated by displacement condition and standard normal force by an universal tester. The measured values by the universal tester were converted by constant elastic modulus when strains were 20% (10 mm square sensor) and 25% (20 mm square sensor). The full scale (FS) error of each force/torque component was calculated by (estimated value – theoretical value) / FS range. Tables 1 and 2 shows FS ranges in each displacement of the 10 and 20 mm square sensors. Table 3 shows FS error of force/torque estimation in the 20 mm square sensor. Column 1 shows maximum values in each force/torque component. The averaged value (column 2) was calculated in each direction of 625 points. Table 4 shows a part of results in Table 3 and shows calculated and theoretical force/torque in five displacement conditions in addition to FS error. 

**Table 3:** FS error of forces and torque estimation in the 20 mm square sensor

**Direction**	**FS error**	**(Unit:%)**
	**Maximum value**	**Average value**
***X***	4.1	1.1
***Y***	6.3	1.8
***Z***	14.1	1.8
***Θ***_**Z**_	16.4	3.9

**Table 4:** **Five conditions in FS error and forces and torque of Table**3

	**Direction**	**Condition**					**Unit**
		**1**	**2**	**3**	**4**	**5**	
**Displacement**	***X***	2	1	0	−1	−2	mm
	***Y***	−2	−1	0	1	2	mm
	***Z***	2	1.6	1.2	0.8	0.5	mm
	***Θ***_**Z**_	−10	−5	0	5	10	degree
**Calculated**	***X***	2.9	1.4	−0.1	−1.5	−3.0	N
**force/torque**	***Y***	−3.0	−1.3	0.0	1.2	2.8	N
	***Z***	8.9	7.1	5.0	3.6	2.2	N
	***Θ***_**Z**_	−17.5	−7.3	0.7	7.8	16.2	N·m
**Theoretical**	***X***	2.9	1.4	0.0	−1.4	−2.9	N
**force/torque**	***Y***	−2.9	−1.4	0.0	1.4	2.9	N
	***Z***	8.7	6.9	5.2	3.5	2.2	N
	***Θ***_**Z**_	−16.8	−8.4	0.0	8.4	16.8	N·m
**FS error**	***X***	0.6	0.6	2.1	0.7	1.5	%
	***Y***	1.5	1.7	0.0	3.6	1.0	%
	***Z***	3.0	2.5	2.1	1.7	0.2	%
	***Θ***_**Z**_	2.2	3.4	2.2	1.7	1.8	%

## Discussion

### Capacitance characteristics of the 10 mm square sensor

In the *X* direction (Figure 6A), the shape could be divided into three types, named convex, increasing, and decreasing types. The convex type had the maximum value at *X* = 0 mm and was observed in the characteristics of FEC and OEC (AD’, BC’, CB’, DA’). The increasing type increased with increasing *X* and was observed in the characteristics of OEC (AB’, DC’) and DEC (AC’, DB’). The decreasing type decreased with increasing *X* and was observed in the characteristics of OEC (BA’, CD’) and DEC (BD’, CA’). The difference of capacitance value in the same type was derived from the difference of initial distance in the combination of upper and lower electrodes. In the point of *X* = −0.2 mm of FEC (AA’, BB’) (arrow in Figure 6A), the corrected value was temporarily diminished by a shift of the maximum value to the negative domain. The shift arose from the position gap between silicone gel and the substrate in the process of assembling the sensor.

In the *Y* direction (Figure 6B), the types of shape were the same as those in the *X* direction. The convex type had the maximum value at *Y* = 0 mm and was observed in the characteristics of FEC and OEC (AB’, BA’, CD’, DC’). The increasing type increased with increasing *Y* and was observed in the characteristics of OEC (AD’, BC’) and DEC (AC’, BD’). The decreasing type decreased with increasing *Y* and was observed in the characteristics of OEC (CB’, DA’) and DEC (CA’, DB’). The shape and amount of capacitance in the *Y* direction (Figure 6B) were similar to the shape and amount in the *X* direction (Figure 6A). The line capacitance which occurred in upper and lower electrode lines was involved in the measured capacitance. The line capacitance in the *Y* direction was different from the line capacitance in the *X* direction because the electrode substrate had a different pattern for each axis (Figure 4A). When displacement *Y* was applied to the sensor, the line capacitance between the electrode lines corresponding to the combination of upper and lower electrodes varied according to the amount of the displacement *Y*. At the arrows in Figure 6B, there was a temporary increase in the corrected capacitances for the effect of the line capacitance between the electrode lines. In BD’, the lower electrode and line D’ moved toward the upper electrode and line B with increasing *Y*. When the distance between lines B and D’ is least in varied *Y*, the line capacitance has a maximum value.

In the *Z* direction (Figure 6C), the capacitances of all combinations of upper and lower electrodes increased exponentially with increasing *Z* because of the decreasing of *d*. The corrected value was nearly identical to the theoretical value.

In the *Θ*_Z_ direction (Figure 6D), the shape could be divided into four types, named convex, concave, increasing, and decreasing types. The convex and concave types had maximum and minimum values at *Θ*_Z_ = 0 degrees and were observed in the characteristics of FEC and DEC. The increasing type increased with increasing *Θ*_Z_ and was observed in the characteristics of OEC (AD’, BA’, CB’, DC’). The decreasing type decreased with increasing *Θ*_Z_ and was observed in the characteristics of OEC (AB’, BC’, CD’, DA’). The amount of *d* change generated by the varied *Θ*_Z_ was the smallest in the amounts of *d* change generated by all varied displacement components. The combinations of FEC only had an overlap area of paired electrodes. Proximity of paired electrodes occurred in other combinations that had no overlap area of paired electrodes. In the methods for calculation of displacement and the calculation of theoretical capacitance, upper and lower electrodes were assumed to be parallel plate type in distance between center points of the paired electrodes. Therefore, measured value contains small error derived from the supposition. And, main factor causing change of capacitance was the occurrence of overlap area and proximity of paired electrodes. In addition, the overlapping effect of the paired electrode line arose at a small yaw angle in all combinations without FEC. For example, an overlapping effect occurred at yaw angle of approximately 7 degree in AD’. The electrode lines of the 10 mm square sensor were connected in ground G, D (D’), C (C’), B (B’), and A (A’) from the left side. The occurrence of proximity of paired electrodes and overlapping of the paired line was divided into the following four patterns.

(i) Proximity of upper and lower electrodes and proximity of upper and lower electrode lines.

(ii) Proximity of upper and lower electrodes and withdrawal of upper and lower electrode lines.

(iii) Withdrawal of upper and lower electrodes and proximity of upper and lower electrode lines.

(iv) Withdrawal of upper and lower electrodes and withdrawal of upper and lower electrode lines.

**Figure 8:**
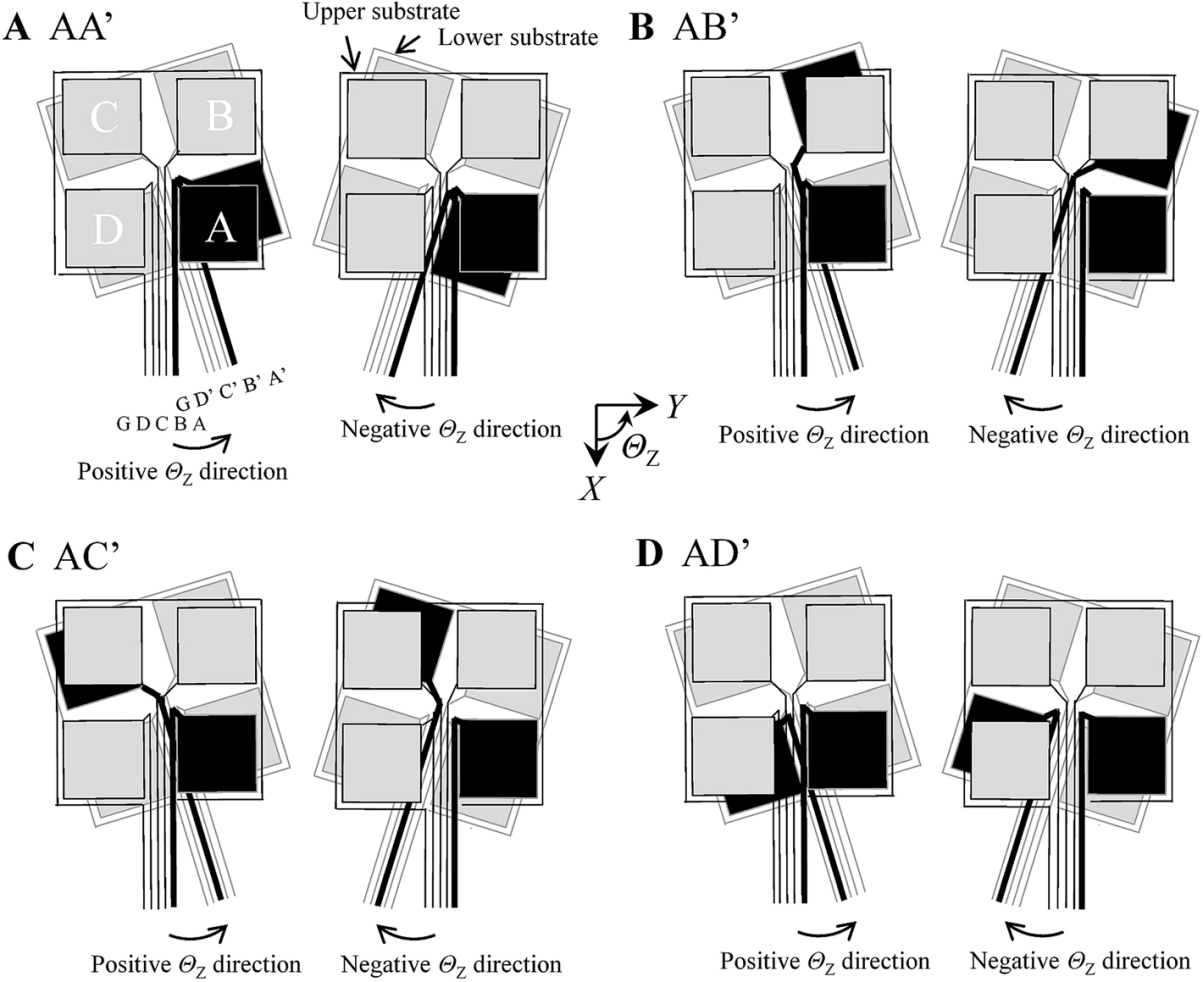
**Movement of paired electrodes and lines (10 mm square sensor). A.** FEC (AA’). **B.** OEC (AB’) **C.** DEC (AC’). **D.** OEC (AD’).

Figure 8 shows an example in FEC(AA’), OEC(AB’), DEC(AC’) and OEC (AD’). The lower substrate moved with varying yaw angle in the same measurement condition. The behaviors in positive/negative domains of *Θ*_Z_ were applicable to (iv)/(iv) in AA’, (iii)/(ii) in AB’, (i)/(ii) in AC’ and (i)/(iv) in AD’. All combinations of paired electrodes coincided with any pattern. Therefore, by the overlapping effect, the measured curve of capacitance had a different tendency than that of the theoretical curve. The measured capacitance was corrected by linear approximation for adjustment of gain and offset. The corrected points in varied *X*, *Y* and *Θ*_Z_ were three points in positive and negative domains, respectively. The amount of line capacitance in each region of displacement is different because of difference in displacement points of maximum line capacitance.

An overlapping effect of lines occurred in the *Y* and *Θ*_Z_ directions. It was thought that capacitance varied in the paired line and the shielded cables connecting the line and LCR meter. Thus, it is important to prevent line capacitance such as the back side ground of the substrate and the shielding of paired electrode lines.

When the measured value includes a nonlinear error, it is necessary for the estimation of displacements in arbitrary points to understand the relation between the displacement and the measured capacitance including the error. In this study, displacements were calculated by the equations of a sphere. However, it is difficult to estimate displacements of arbitrary points by this calculation method because the amount of correction has to be determined for obtaining appropriate capacitance in advance. We have therefore developed an iterative calculation method that can be used to estimate displacements of arbitrary points in the allowable range of the sensor because the relation of capacitance and displacements in arbitrary points without calibration points was interpolated using measured input–output characteristics. Each displacement is estimated in series within 25 steps of iteration.

### Calculation of force and torque components of the 10 mm square sensor

The standard force measured by a universal tester and the measured value was converted in *E* = 25.7 kPa. The standard force was approximately 1.5 N in the condition of *Z* = 3 mm. *E* was obtained in a test of silicone gel (“Methods, Sensor design and fabrication”) and it was calculated in the strain of 20%. The calculated normal force *F*_Z_ in the same condition was also about 1.5 N (Figure 7C). Therefore, it was thought that the calculated force was appropriate. The FS error was calculated from the absolute error between estimated and theoretical values of force and torque components. FS of force/torque is shown in Table 1. FS error in Figure 7 was the maximum value in force and torque components of each varied displacement. The range of maximum FS errors was 0.4-10.1%. The FS error increased at the measured point affected by the position gap between the substrates and the overlapping of lines corresponding to the paired electrodes. The error ratios in the force and torque calculation were equal to those in the displacement calculation because the force and torque are proportional to the strain as shown in the section “Sensor theory, Calculation of force and torque components”. In this result, standard normal force was converted in a constant elastic modulus. Actually, however, normal force varies with increasing displacement *Z* in a nonlinear fashion for change in stiffness. An iterative calculation method for estimation of force enables estimation of force that has a nonlinear characteristic in a manner similar to the iterative calculation method of displacements.

### Calculation of force and torque components of the 20 mm square sensor

**Figure 9:**
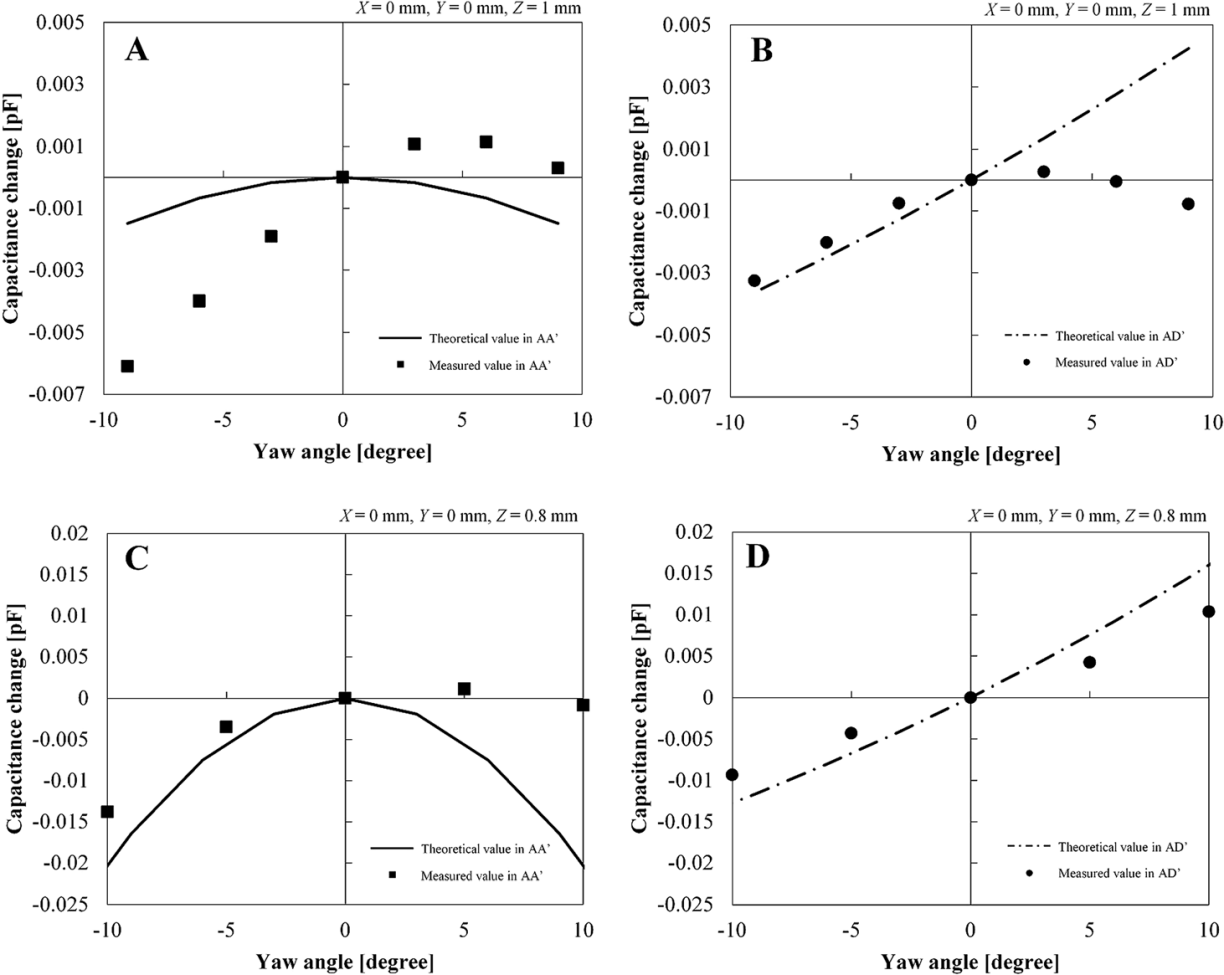
**Overlapping effect of paired electrode line in capacitance characteristics.A.** AA’ of the 10 mm square sensor. **B.** AD’ of the 10 mm square sensor. **C.** AA’ of the 20 mm square sensor. **D.** AD’ of the 20 mm square sensor.

A new sensor of different size (cross sectional area of 20 mm square) was developed and the capacitance was measured in four DOF displacements. As shown in Figure 4C, we improved the electrode pattern and grounding means of electrode lines in consideration of the overlapping effect in the results obtained for the 10 mm square sensor. The upper and lower electrode lines in the 20 mm square sensor were not facing each other, and the electrode line connected to the ground area was placed in the middle. The electrode lines were connected in D (D’), C (C’), ground G, B (B’), and A (A’) from the left side. The electrode lines were shrouded in pressure-sensitive adhesive tape for electrical insulation (Kempton tape, P-221, Permacel) and a stainless plate connected to ground was located between the upper and lower electrode lines. Figure 9 shows the overlapping effects of AA’ and AD’ in the 10 and 20 mm square sensors. The horizontal axis is the varied yaw angle and the vertical axis is the capacitance change that is differential value at the value of *Θ*_Z_ = 0 degree. Gaps of peaks in AA’ were derived from the position gap of the upper and lower substrates in the process of the assembling the sensor. In AD’ of the 10 mm square sensor, the capacitance curve in the positive domain of yaw angle was decreased compared to the theoretical value for withdrawal of electrode lines A and D’. The overlapping condition of the lines occurred in approximately 3 degree. In AD’ of the 20 mm square sensor, the line effect was diminished by the improvement. The displacements and forces were calculated in the same way as that for the 10 mm square sensor.

In estimation in force/torque of four DOF, as shown in Table 3, maximum FS errors through 625 measured points were 4.1%, 6.3%, 14.1% and 16.4% in *X*, *Y*, *Z* and *Θ*_Z_, and average errors were 1.1%, 1.8%, 1.8% and 3.9%. Although the overlapping effect of line capacitance was remedied by improvement of the electrode pattern and grounding means of electrode lines, position gap of upper and lower electrodes occured. Most of the calculated normal forces in the condition of *Z* = 2 mm were approximately 8.6 N. The standard normal force that was converted in *E* = 54.1 kPa was 9.4 N in the condition of *Z* = 2 mm. *E* was newly measured using silicone gel of 20 mm×20 mm×5 mm and it was calculated in the strain of 25% of silicone gel. In the 20 mm square sensor, stiffness of the silicone gel was greater than that in the 10 mm square sensor. Therefore, the range of detection of yaw angle in the 20 mm square sensor was narrower than that in the 10 mm square sensor. In real measurements for subjects, motion of subjects at high risk for pressure ulcers is decreased by restraint of spontaneous movement. Thus, it is considered that the yaw angle range of the 20 mm square sensor was sufficient to measure rotation applied to skin. Table 4 shows examples in Table 3. We considered the accuracy to be within an acceptable range.

### Sensor configuration and materials for real measurement

The sensor deforms along skin surface of the subject because of flexibility of upper and lower substrates. To prevent the deformation of upper and lower substrates, thin and stiff plates are affixed on the substrates for homogenization of applied force. If one substrate is tilted to the other substrate, the measured value includes the effects of pitch and roll torques. In this situation, upper and lower electrodes are assumed to be parallel plate type in distance between center points of the paired electrodes in the method for calculation of displacement. The developed sensor enables detection of torque components *T*_X_ and *T*_Y_. However, because the developed sensor does not have the ability for detection of pitch and roll torques, the measurement in the situation should be avoided. Therefore, it is important to arrange an appropriate surface on a bony prominence and to confine motion of the subject during measurement.

Appropriate selection of materials is important for mitigation of error generated by different materials and consideration for the subject [9]. The creep of silicone gel was tested to select an appropriate material for measurement of force applied to the skin surface. The selection of silicone gel was determined on the basis of appropriate *E* and *RC*. Double-sided tape was selected on the basis of flatness, thin thickness and adhesion strength. The thickness of the double-sided tape was 0.085 mm. The adhesion strength was good when shear displacements and yaw angle were applied to the sensor. In fact, the developed sensor is different from the theoretical condition for a stacked material (silicone gel, double-sided tape and flexible substrate). We considered that the change in structural stiffness by double-sided tape and substrate is small because these materials were thin. However, the capacitance is affected according to the change in thickness of double-sided tape. And, the developed sensor is covered by a protective material such as a polyurethane film after thin and stiff plates are established in upper and lower substrates. An appropriate protective material needs to have the ability of flexibility for detection of yaw angle. The protection may have an undesirable influence on the detection range of detection of displacements.

An error may be observed by motion artifact during measurement for one condition. It is possible to diminish the effect by averaging of several measured points in the same condition. The signal processing circuit for real measurement is still in the trial phase. Time constant and delay in measurement are determined by the circuit and developed sensor. In the displacement estimation method under development, the number of iterations in a calculation for one condition was 25. It is considered to be a sufficiently short time. However, if several measured points in the same condition are measured for decreasing the effect of motion artifact, the time for total measurement is extended. In real measurement, the sensor is arranged on mattress before the subject take target body position. Then, force/torque is measured in appropriate surface on bony prominence. A sequence of actions may be a heavy work for the subject. And, nonrestraint during measurement is desirable for the subject as indicated by [15]. However, the motion of the subject is limited during measurement in our sensor.

## Conclusions

The goal of our study was to develop a novel capacitive force sensor that enables simultaneous measurements of yaw torque around the pressure axis and normal force and two orthogonal shear forces for the purpose of elucidation of pressure ulcer pathogenesis and establishment of criteria for selection of cushions and mattresses. In this paper, we described the fabrication of and measurement using a prototype sensor and the validity of forces calculated by a method for estimation based on the sensor structure. Two types of prototype sensors were constructed from cubic silicone gel (10 mm×10 mm×5 mm and 20 mm×20 mm×5 mm) as a dielectric material and four upper and lower electrodes (3.5 mm×3.5 mm and 7 mm×7 mm) which were established at two electrode substrates of FPCB, respectively. There are sixteen combinations of upper and lower electrodes, and the paired electrode have the function of a capacitive parallel plate type for the detection of force and torque components. Capacitances of sixteen capacitors were measured in the displacement ranges of 0–3 mm, –3-3 mm and −30-30 degrees (10 mm square sensor) and 0–2 mm, –2-2 mm and −10-10 degrees (20 mm square sensor) in pressure direction, shear directions and rotation direction around the pressure axis instead of forces and torque of 0–1.5 N, –0.5-0.5 N and −1.5-1.5 N mm (10 mm square sensor) and 0–8.7 N, –2.9-2.9 N and −16.8-16.8 N mm (20 mm square sensor) in normal force, shear forces and yaw torque, respectively. The force and torque components at the measured capacitance point were calculated by corrected capacitance. The calculated normal force was equal to the theoretical value and standard normal force measured by a universal tester (1.5 N) in the condition of *Z* = 3 mm (10 mm square sensor). In the 20 mm square sensor, the calculated normal force was 8.6 N, theoretical normal force was 8.7 N and standard normal force was 9.4 N in the condition of *Z* = 2 mm. Therefore, it was thought that the calculated force was appropriate. The repeatability was 3.23% in all capacitance measurements in the 10 mm square sensor. The FS errors of force and torque components calculated according to the corrected capacitances were less than or equal to 10.1% and 16.4% in the 10 and 20 mm square sensors, respectively. We considered accuracy to be within an acceptable range. As a future work, improvements to the appropriate ground procedure and the position gap in fabrication of the sensor will be made. Furthermore, the newly developed sensor has a structure that enables detection of roll and pitch torque components around the orthogonal axis. We have developed an iterative calculation method corresponding to the multi DOF to directly estimate displacement from the measured value including an error.

## Competing interests

The authors declare that they have no competing interests.

## Authors’ contributions

CM measured and validated data and engaged in fabrication of the sensor. YI selected the material and fabricated the sensor and also measured and collected data. MT checked the data and mentored in all processes. All authors read and approved the final manuscript.

## References

[B1] Ministry of Health Labour and Welfare White Paper on Health, Labour and Welfare 2012 2012 Japan: Nikkei Printing In Japanese

[B2] Hulsenboom MA Bours GJ Halfens RJ Knowledge of pressure ulcer prevention: a cross-sectional and comparative study among nurses BMC Nurs 2007 6 2 10.1186/1472-6955-6-2 17349049 PMC1821326

[B3] Salcido R Popescu A Ahn C Animal models in pressure ulcer research J Spinal Cord Med 2006 30 2 107 116 17591222 10.1080/10790268.2007.11753921 PMC2031948

[B4] Ohura T Takahashi M Ohura N Jr Influence of external forces (pressure and shear force) on superficial layer and subcutis of porcine skin and effects of dressing materials: are dressing materials beneficial for reducing pressure and shear force in tissues? Wound Repair Regen 2008 16 1 102 107 10.1111/j.1524-475X.2007.00325.x 18086290

[B5] Leilnahari K Fatouraee N Khodalotfi M Sadeghein MA Kashani YA Spine alignment in men during lateral sleep position: experimental study and modeling Biomed Eng Online 2011 10 103 10.1186/1475-925X-10-103 22129355 PMC3265433

[B6] Akins JS Karg PE Brienza DM Interface shear and pressure characteristics of wheelchair seat cushions J Rehabil Res Dev 2011 48 3 225 234 10.1682/JRRD.2009.09.0145 21480097

[B7] Davis BL Foot ulceration: hypotheses concerning shear and vertical forces acting on adjacent regions of skin Med Hypotheses 1993 40 1 44 47 10.1016/0306-9877(93)90195-V 8455466

[B8] Chao LP Chen KT Shape optimal design and force sensitivity evaluation of six-axis force sensors Sensor Actuator Phys 1997 63 2 105 112 10.1016/S0924-4247(97)01534-3

[B9] Ciaccio EJ Hiatt M Hegyi T Drzewiecki GM Measurement and monitoring of electrocardiogram belt tension in premature infants for assessment of respiratory function Biomed Eng Online 2007 6 13 10.1186/1475-925X-6-13 17445262 PMC1868740

[B10] Wang L Beebe DJ Characterization of a silicon-based shear-force sensor on human subjects IEEE Trans Biomed Eng 2002 49 11 1340 1347 10.1109/TBME.2002.804586 12450364

[B11] Hwang ES Seo JH Kim YJ A polymer-based flexible tactile sensor for both normal and shear load detections and its application for robotics J Microelectromech Syst 2007 16 3 556 563

[B12] da Rocha JGV da Rocha PFA Lanceros-mendez S Capacitive sensor for three-axis force measurements and its readout electronics IEEE Trans Instrum Meas 2009 58 8 2830 2836

[B13] Cheng MY Lin CL Lai YT Yang YJ A polymer-based capacitive sensing array for normal and shear force measurement Sensors 2010 10 11 10211 10225 10.3390/s101110211 22163466 PMC3230994

[B14] Yusuke I Basic study of a force sensor that enables simultaneous measurement of normal/shear forces and torque. MD thesis 2009 Graduate School of Information Science and Technology: Hokkaido University In Japanese

[B15] Ciaccio EJ Drzewiecki GM Tonometric arterial pulse sensor with noise cancellation IEEE Trans Biomed Eng 2008 55 10 2388 2396 18838364 10.1109/TBME.2008.925692

